# Comparative genomics of the emerging human pathogen *Photorhabdus asymbiotica *with the insect pathogen *Photorhabdus luminescens*

**DOI:** 10.1186/1471-2164-10-302

**Published:** 2009-07-07

**Authors:** Paul Wilkinson, Nicholas R Waterfield, Lisa Crossman, Craig Corton, Maria Sanchez-Contreras, Isabella Vlisidou, Andrew Barron, Alexandra Bignell, Louise Clark, Douglas Ormond, Matthew Mayho, Nathalie Bason, Frances Smith, Mark Simmonds, Carol Churcher, David Harris, Nicholas R Thompson, Michael Quail, Julian Parkhill, Richard H ffrench-Constant

**Affiliations:** 1 School of Biosciences, University of Exeter in Cornwall, Penryn, TR10 9EZ, UK; 2 Department of Biology and Biochemistry, University of Bath, Bath, BA2 7AY, UK; 3 Pathogen Sequencing Unit, The Wellcome Trust Sanger Institute, Hinxton, Cambridge, CB10 1SA, UK

## Abstract

**Background:**

The Gram-negative bacterium *Photorhabdus asymbiotica *(Pa) has been recovered from human infections in both North America and Australia. Recently, Pa has been shown to have a nematode vector that can also infect insects, like its sister species the insect pathogen *P. luminescens *(Pl). To understand the relationship between pathogenicity to insects and humans in *Photorhabdus *we have sequenced the complete genome of Pa strain ATCC43949 from North America. This strain (formerly referred to as *Xenorhabdus luminescens *strain 2) was isolated in 1977 from the blood of an 80 year old female patient with endocarditis, in Maryland, USA. Here we compare the complete genome of Pa ATCC43949 with that of the previously sequenced insect pathogen *P. luminescens *strain TT01 which was isolated from its entomopathogenic nematode vector collected from soil in Trinidad and Tobago.

**Results:**

We found that the human pathogen Pa had a smaller genome (5,064,808 bp) than that of the insect pathogen Pl (5,688,987 bp) but that each pathogen carries approximately one megabase of DNA that is unique to each strain. The reduced size of the Pa genome is associated with a smaller diversity in insecticidal genes such as those encoding the Toxin complexes (Tc's), Makes caterpillars floppy (Mcf) toxins and the *Photorhabdus *Virulence Cassettes (PVCs). The Pa genome, however, also shows the addition of a plasmid related to pMT1 from *Yersinia pestis *and several novel pathogenicity islands including a novel Type Three Secretion System (TTSS) encoding island. Together these data suggest that Pa may show virulence against man via the acquisition of the *pMT1*-like plasmid and specific effectors, such as SopB, that promote its persistence inside human macrophages. Interestingly the loss of insecticidal genes in Pa is not reflected by a loss of pathogenicity towards insects.

**Conclusion:**

Our results suggest that North American isolates of Pa have acquired virulence against man via the acquisition of a plasmid and specific virulence factors with similarity to those shown to play roles in pathogenicity against humans in other bacteria.

## Background

Comparative genomics has been widely used to explain differences in the lifestyle and pathogenicity of different bacteria, ranging from *Burkholderia *[1] to *Yersinia *[2]. We are interested in the shift in lifecycle between bacteria that are insect pathogens, or are insect-associated, and the emergence of pathogenicity to man [3]. Here we study the *Photorhabdus *group of bacteria which are associated with entomopathogenic nematodes (EPNs) and their insect hosts [4]. Both the species *P. luminescens *(Pl) and *P. temperata *(Pt) have only ever been recovered from EPNs isolated from infected insect hosts. Until recently, however, the other species *P. asymbiotica *(Pa) has only been recovered as clinical isolates from human wounds, either in North America or Australia [5,6]. We have therefore chosen to sequence the genome of one of the North American clinical isolates of *P. asymbiotica *strain ATCC43949 (formerly described as *Xenorhabdus luminescens *strain 2 [5]), in order to compare it with the previously sequenced insect pathogen *P. luminescens *TT01 recovered from its nematode vector, collected by baiting soil with insects, in Trinidad and Tobago. Recent comparative studies between the genome of Pl TT01 and *Y. enterocolitica *have highlighted the genes within Pl that are likely to be insecticidal [7], and here we identify genomic changes associated with the additional selective pressures exerted when *Photorhabdus *meets the vertebrate immune system for the first time.

The lifecycle of *Photorhabdus *bacteria is complex and requires the recognition of, and association with, a large variety of biological substrates and hosts, specifically nematodes of the genus *Heterorhabditis*, a wide variety of insects and now with the emergence of Pa, man. Bacteria of the species Pl or Pt, live in a strict association with specific strains of EPN. This association is even more complicated than originally anticipated [8], as the bacteria associate with both the hermaphroditic adult and the infective juveniles (IJs) that emerge from the inside of the adults themselves (see Results for detailed discussion). The net result of the nematode lifecycle is that the adults sacrifice themselves as food for the developing juveniles, which grow within the hemocoel of the mother, and the IJs then re-associate with the bacteria leave the insect cadaver and invade new insect hosts. The IJs swim in the soil water and actively seek out and penetrate insect hosts. Once inside the insect, the IJs regurgitate the *Photorhabdus *bacteria (50–250 cells) that are held within the IJ's gut [9]. The bacteria then resist the insect immune system [10,11] and release a lethal cocktail of virulence factors [12] that kill the insect and render it as a suitable food source for the bacteria. In turn, the developing nematodes feed off the multiplying bacteria until feeding is halted and nematode reproduction starts. As the insect host must die in order for the lifecycle to be completed, sequencing of the Pl TT01 genome has revealed more toxin encoding genes than any other bacterial genome to date. This plethora of toxin genes is speculated to represent functional redundancy or 'over-kill' in order to ensure the death of a wide range of insect hosts [13].

Different strains of Pl or Pt are traditionally recovered by baiting soil samples with insect larvae, watching for larvae that become infected and glow in the dark, and finally isolating the bacteria from the insect cadaver. *Photorhabdus *is the only terrestrial bacterium with a *lux *operon [14] and the infected insects therefore emit light generated by the infecting bacteria. In stark contrast, strains of Pa have, until recently, only ever been recovered from human wounds, either in North America [5] or on the Gold Coast of Australia [6]. Following the recent infection of a man digging a fence post hole with his hand in sandy soil at Kingscliffe on the Australian Gold coast, we reasoned that we were likely to recover a Pa nematode vector by baiting soil from the same fence post hole with insect larvae. This was indeed the case and a new species of *Heterorhabditis *nematode has been recovered that contained exactly the same strain of Pa as that recovered from the patient's hand [15]. This story demonstrates that all Pa strains are likely to have EPN vectors and that the colonization of man is a novel and specific part of the Pa lifecycle. Although a nematode vector has not been confirmed for Pa isolates from North America, the simplest hypothesis is that Pa is vectored by nematodes in both North America and Australia. To look for genes potentially lost or gained upon this novel host switch, here we present the complete genome sequence of a North American strain of Pa, strain ATCC43949. This strain was isolated in 1977 from a female patient with endocarditis in Maryland, USA, and was originally referred to as *Xenorhabdus luminescens *strain 2 from DNA hybridization group 5 [5]. We compare this Pa strain with the Pl strain TT01, isolated from its vector nematode collected from an insect used to bait soil in Trinidad and Tobago [16]. We note that, in common with all other Pa strains, ATCC43949 has acquired a plasmid, perhaps important in virulence against man. We also discuss the loss of insecticidal genes and the gain of novel secretion systems and effectors that other groups of bacteria use in virulence against vertebrate hosts.

## Results

### Only Pa strains carry plasmids

**Figure 1:**
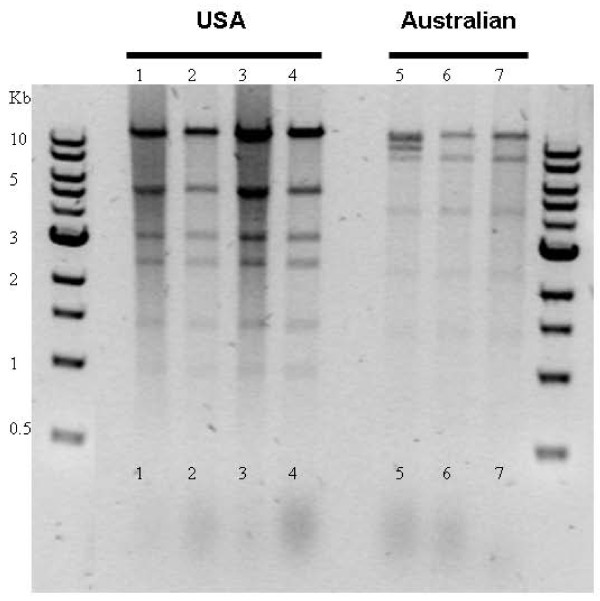
**Strains of *P. asymbiotica*, either from the USA or Australia, are the only *Photorhabdus *that carry plasmids**. Restriction enzyme (*Bgl*2) analysis of Pa plasmids confirms the similarity of plasmids in different USA (lanes 1–4) and Australian (lanes 5–7) isolates. Strains are from North America: 1, ATCC43950 (San Antonio, Texas); 2, ATCC43951 (San Antonio, Texas); 3; ATCC43952 (San Antonio, Texas); 4, ATCC43949 (Maryland) and Australia: 5, Beaudesert (Queensland), 6, Murwillumbah (New South Wales) and 7, Gladstone (Queensland). See Gerrard *et al*. 2004 reference [6] for more details of Australian strains and their collection.

**Figure 2:**
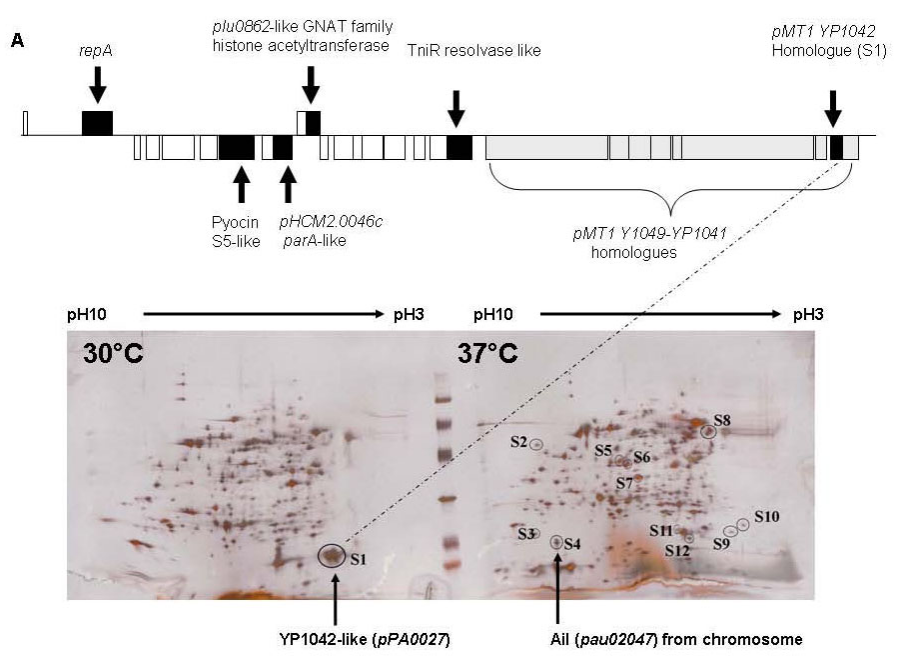
**(A) Predicted open reading frames in the *pPAU1 *circular plasmid sequenced from Pa ATCC43949 presented in a linear diagram**. Most of the open reading frames on the plasmid predict transposases with high similarity to those found in *Y. pestis *(see Table 1) (B) Two-dimensional SDS-PAGE gel of Pa supernatants grown at 30°C (left panel) and 37°C (right panel). Proteins that are differentially expressed are circled and labelled S1-S12. Analysis of proteins S1-S12 by tryptic digest and comparison to a database of predicted tryptic digest fragments from predicted Pa proteins identified S1 as YP1042-like and S4 as Ail-like. The fully annotated sequence of *pPAU1 *is GenBank accession number AC FM162592.

The acquisition of plasmids is often a mechanism of increasing the mammalian pathogenicity of a bacterium, for example one of the central differences between *Yersinia pseudotuberculosis *and the highly pathogenic *Y. pestis *is the acquisition of plasmids involved in flea vectoring and evasion of the mammalian immune system [17]. We therefore examined the Pa strains we have collected to date for the presence of plasmids. All of the Pa strains examined, either from North America or Australia, carry plasmids (Figure 1), whereas no other *Photorhabdus *strain we have examined from the two other main groups, Pl and Pt, shows the presence of any plasmid (data not shown). In the genome sequencing of Pa ATCC43949 the 29,732 bp plasmid (GenBank accession number AC FM162592), here termed *pAU1*, was the first complete circular element to assemble (Figure 2A). Restriction enzyme analysis of plasmids in four other North American Pa strains suggests that they all carry this same plasmid (Figure 1). BLAST analysis of *pAU1 *reveals few open reading frames predicting known proteins but reveals an extensive array of transposons similar to those found in the genome and plasmids of *Y. pestis *(Table 1) To date we have not been able to ascribe firm biological functions to the proteins predicted by *pAU1 *coding regions (CDs). However, two-dimensional gel and proteomic analysis of Pa cultures grown at different temperatures (Figure 2B) has shown the small protein encoded by the *pMT1 Y1042*-like gene is highly secreted into the supernatant during growth at 30°C but not 37°C, suggesting that its expression is detrimental in the mammalian host. Interestingly, Pl TT01 harbouring *pPAU1 *marked with a tetracyclin resistance transposon, shows a reduced ability to grow in Luria Broth (LB) media. At this point it is not clear if this reduced growth represents a problem with plasmid replication in Pl or whether *pPAU1 *encodes factors toxic or incompatible with Pl metabolism or gene regulation. Two *pPAU1 *genes that could set up such an incompatibility include the GNAT family histone acetyltransferase which may alter global gene expression and an S5 pyocin-like gene and its immunity protein encoding gene which might act as a plasmid stabilization mechanism.

**Table 1:** Annotated proteins in the *P. asymbiotica *AC FM162592 plasmid

**pPAU1 plasmid CDS**	**Protein**	**Organism**	**Score**	**E-value**
*pPA0001*	IS21 family transposase	*Yersinia pestis Angola*	357	8.00E-097
*pPA0002*	hypothetical protein YPMT1.86c – putative transposase	*Yersinia pestis *CO92	101	1.00E-019
*pPA0003*	putative integrase	*Yersinia pestis *FV-1	331	6.00E-089
*pPA0004*	hypothetical protein	*Pasteurella pneumotropica*	43.5	0.035
*pPA0005*	hypothetical protein PROPEN_02818	*Proteus penneri *ATCC 35198	206	4.00E-051
*pPA0006*	hypothetical protein HCM2.0001c	*Salmonella enterica *subsp *enterica *serovar Typhi str. CT18	66.2	5.00E-009
*pPA0007*	replication protein repA	*Escherichia coli*	220	2.00E-055
*pPA0008*	hypothetical protein plu0861	*Photorhabdus luminescens *subsp. *laumondii *TTO1	162	4.00E-038
*pPA0010*	hypothetical protein plu0862	*Photorhabdus luminescens *subsp. *laumondii *TTO1	295	5.00E-078
*pPA0011*	NO HITS	NO HITS		
*pPA0012*	NO HITS	NO HITS		
*pPA0013*	replication protein repA	*Escherichia coli*	342	5.00E-092
*pPA0014*	NO HITS	NO HITS		
*pPA0016*	replication protein repA	*Escherichia coli*	342	5.00E-092
*pPA0017*	hypothetical protein HCM2.0001c	*Salmonella enterica *subsp. *enterica *serovar Typhi str. CT18	114	2.00E-023
*pPA0018*	resolvase	*Yersinia pestis *biovar *Orientalis *strain IP275	236	4.00E-060
*pPA0019*	hypothetical protein Gura_3302	*Geobacter uraniireducens *Rf4	370	2.00E-100
*pPA0020*	transposase	*Yersinia pestis Antiqua*	1561	0
*pPA0021*	putative transposase	*Yersinia pestis *CO92	149	5.00E-034
*pPA0022*	hypothetical protein pG8786_001	*Yersinia pestis*	340	1.00E-091
*pPA0023*	transposase	*Yersinia pestis Pestoides *F	365	5.00E-099
*pPA0024*	putative transposase	*Yersinia pestis *CO92	117	2.00E-024
*pPA0025*	putative transposase	*Yersinia pestis *CO92	860	0
*pPA0026*	transposase	*Yersinia pestis Antiqua*	112	8.00E-023
*pPA0027*	putative transposase	*Yersinia pestis *CO92	124	2.00E-026
*pPA0028*	putative transposase	*Yersinia pestis *CO92	233	3.00E-059
*pPA0029*	putative transposase	*Yersinia pestis *CO92	52.8	6.00E-005

### Genome sequencing of Pa ATCC43949 and synteny with Pl TT01

**Figure 3:**
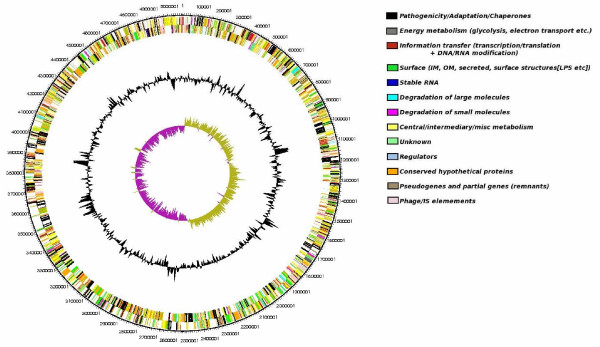
**Schematic circular diagram of the single 5,064,808 bp chromosome of Pa ATCC43949**. The circles show (from outside to inside): 1, DNA coordinates (black); 2, CDSs colour coded as to function (black, pathogenicity/adaptation; dark grey, essential metabolism; red, DNA replication, transcription and restriction modification; green, transmembrane/outer membrane; cyan and magenta, degradation of large and small molecules respectively; yellow, intermediary metabolism; light green, hypothetical; light blue, regulators, orange, conserved hypothetical; brown, pseudogenes; pink, transposons and phage); 3, GC skew and 4, GC deviation. The fully annotated sequence of Pa ATCC43949 is GenBank accession number AC FM162591.

**Figure 4:**
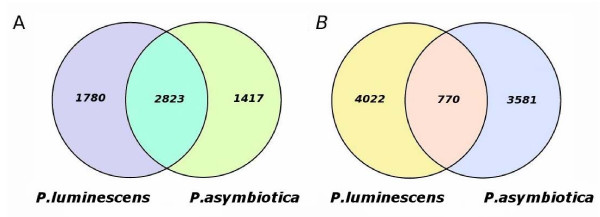
**Venn diagrams showing the results of a BLASTCLUST analysis of orthologous CDS between Pa and Pl**. (A) Numbers of orthologs with 75% identity and (B) numbers of orthologs at 95% identity.

**Figure 5:**
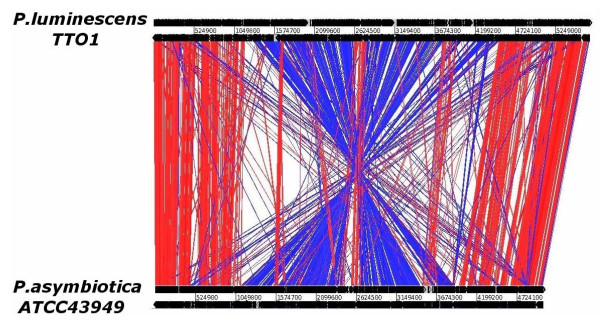
**Alignment of the genomes of Pl TT01 and Pa ATCC43949 using the ARTEMIS comparison tool**. Note the broad linear alignment of the two genomes with the presence of several large inversions. The breakpoints of these chromosomal rearrangements are flanked by numerous transposons and directly repeated sequences.

Genomic DNA from Pa ATCC43949 was sequenced by a shotgun approach and sequences were assembled using the Phusion assembler [18], based on read pair information. Finishing the genome was complicated by the presence of numerous direct repeats in the Pa genome and the last gap to be closed corresponds to two nearly identical regions of phage in tandem duplication (*pau02464*-*pau03092 *and *pau03573-pau03610*). The final sequence had an average of 11× coverage across the genome. The Pa genome was compared to Pl using the Artemis comparison tool which allows an interactive visualisation of comparisons between complete genome sequences and associated annotations [19]. The genome of Pa comprises a single circular chromosome of 5,064,808 bp (GenBank accession number AC FM162591) with a total of 4,403 predicted protein-encoding open reading frames which can be classified based on predicted function (Figure 3). We have designated these ORFs with *pau *numbers (*pau00001-04403*) in reference to *Photorhabdus asymbiotica *USA isolate or *pau*. The genome of Pa is significantly smaller than the genome of the strict insect pathogen Pl TT01 which is 5,688,987 bp with 4,905 predicted ORFs (Table 2) but the GC content remains similar (42.2% versus 42.8% for Pa and Pl respectively). To assess the level of similarity between the predicted proteins from each genome we performed a BLASTCLUST analysis on the two genomes (Figure 4). At the level of 95% identity only 770 predicted proteins are similar between the two genomes but this number increases to 2,823 predicted proteins at the 75% identity level, suggesting that orthologous CDSs are similar yet significantly divergent between the two species groups. Functional classification (Table 3) of the coding sequences in the Pa genome shows that significant percentages of the ORFs encode proteins potentially involved in pathogenicity and adaptation (7.4%), DNA replication, transcription and restriction modification (7.5%) and mobility (transposons/phage) (6.7%). To assess synteny between the Pa and Pl genomes we aligned the two genomes in the comparison tool ARTEMIS comparison tool ACT (Figure 5). The genomes of Pa and Pl show strong synteny across much of their length, however there are several large scale (>10 Kb) inversions in the central regions of the chromosomes associated with numerous transposons and repeat sequences.

**Table 2:** Comparison of genome statistics for Pa and Pl

**Feature**	***P. asymbiotica ATC43949***	***P. luminescens TTO1***
Chromosome size (base pairs)	5,064,808	5,688,987
G+C(%)	42.2	42.80
Average CDS length	956	985
Protein-coding sequences (CDS)	4403	4905
Conserved with assigned function	2678 (60.82%)	2666 (54.35%)
Conserved with unknown function	1725 (39.18%)	2239 (45.63%)
tRNA genes	81	85

**Table 3:** Classification by function of the ORFs within the genome of Pa ATCC43949

**Function**	
Pathogenicity/adaptation	*325 (7.4%)*
Energy metabolism	*64 (1.5%)*
DNA replication/transcription/restriction modification	*329 (7.5%)*
Transmembrane/outer membrane	*528 (12%)*
Stable RNA	*49 (1.1%)*
Degradation of large molecules	*73 (1.6%)*
Degradation of small molecules	*72 (1.6%)*
Central/Intermediary/miscellaneous metabolism	*797 (18.1%)*
Hypothetical protein	*1733 (39.28%)*
Regulation	*135 (3%)*
Pseudogenes	*2 (0.05%)*
Transposons and phage	*296 (6.7%)*

### A lower diversity of insecticidal toxins in Pa

**Figure 6:**
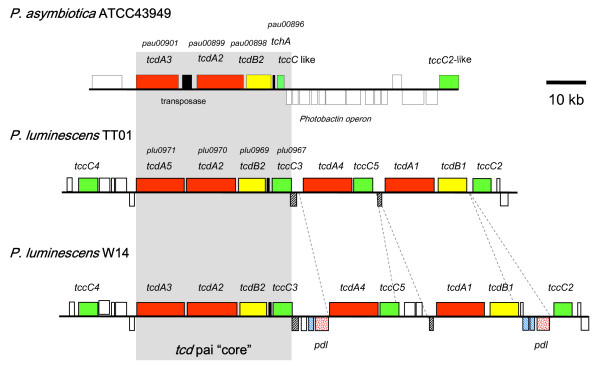
**Diagram comparing the *tcd *islands of Pa ATCC43949 with those from two different strains of Pl: TT01 and W14**. The *tcd *island of Pa appears to represent a conserved 'core' of genes found in all strains whilst both Pl strains contain additional copies of *tcdA*-like, *tcdB*-like and *tccC*-like genes inserted adjacent to this core. Note also that the *tcd *island of Pl W14 has gained several copies of *pdl *genes that encode lipases thought to be responsible for the release of mature W14 Tc complexes into the bacterial supernatant.

**Figure 7:**
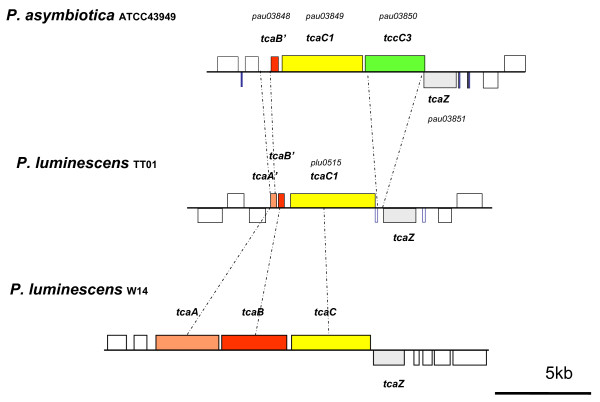
**Diagram comparing the *tca *islands of Pa ATCC43949 with those from Pl TT01 and Pl W14**. Note the presence of all three A, B and C elements (*tcaA, tcaB *and *tcaC*) in the *tca *island of Pl W14 which are required for full oral insecticidal activity in the bacterial supernatant and that the *tcaA *and *tcaB *genes in both Pa ATCC43949 and Pl TT01 have been either deleted or truncated.

**Figure 8:**
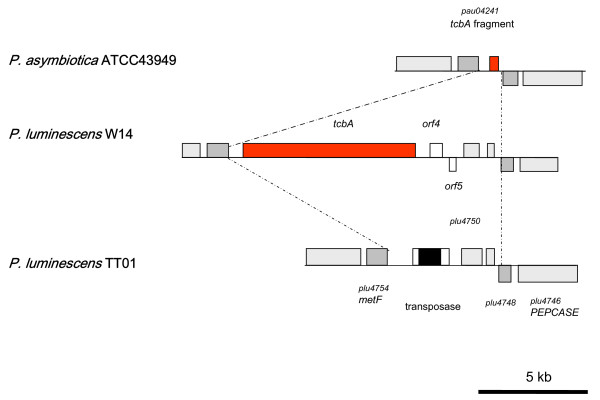
**Diagram comparing the *tcb*-island of Pa ATCC43949 with those from Pl TT01 and Pl W14**. Note that Pl W14 has an intact island with a complete copy of the *tcbA *gene, whereas *tcbA *is either deleted or largely truncated from the two other strains (see Figure 7).

When the Pl TT01 genome was first sequenced, it was described as having more genes encoding toxins than any other genome sequenced to date [16]. Part of the reason that the genome of the emerging human pathogen Pa ATCC43949 is smaller than that of Pl TT01 is a lower number of various genes encoding insecticidal toxins (Table 4). The *toxin complex *(*tc*) genes encode high molecular weight, multi-subunit orally insecticidal toxins. The different Tc's are encoded at discrete locations (pathogenicity islands) in the *Photorhabdus *genome where multiple *tc *gene copies are found. In Pa, the islands encoding the Tca and Tcd-like toxins both encode fewer orthologs. In the Pa *tcd *island, while the "core" region (consisting of *tcdA3*, *tcdA3*, *tcdB2 *and *tccC3*) is present, four *tc *genes, *tcdA1, tcdA4, tcdB1 *and *tccC5*, are absent relative to Pl (Figure 6). In this case it is likely that these extra genes were never acquired by the *tcd*-island of the ancestral strain. In contrast, in the Pa *tca*-island, all of *tcaA *and most of *tcaB *are deleted but a new *tccC *homologue has been acquired (Figure 7). The deletion of *tcaAB *is also seen in Pl TT01, while its close relative Pl W14 maintains an intact and functional *tca *operon. The acquisition of a *tccC *gene next to the intact *tcaC *in Pa creates a "BC" pair which we have previously shown to constitute a functional toxic "unit", even in the absence of TcdA/TcaAB proteins [20]. Previous deletion analysis of the four islands *tca, tcb, tcc *and *tcd *of Pl W14 showed that the loss of oral toxicity to *Manduca sexta *larvae was associated with disruption of either the *tca *or *tcd *islands [21]. We have confirmed that the absence of certain *tc *gene orthologs in these two islands in the emerging human pathogen Pa (Figures 6 and 7), is associated with a lack of oral insecticidal toxicity of both the bacterial cells and culture supernatants. Indeed, the oral toxicity phenotypes of the cells and supernatants of the Pl W14, Pl TT01 and Pa ATCC43949 strains correlates well with the presence and absence of the highly secreted *tcaAB *and the putatively cell surface associated *tcdA1B1*orthologue gene products [22]. Similarly, the copy of *tcbA *is largely deleted from the *tcb*-locus in Pa ATCC43949 (Figure 8), although the role of the Tcb toxin in insecticidal activity is unclear. There are seven *tccC *paralogs in the Pl TT01 genome and Pa seems to have lost some but gained others (Table 4). This is consistent with *tccC *genes being mobile *rhs*-like elements that can readily move around the genome where they often settle next to other *tc *loci [23]. The precise role of TccC is again unclear but TccC is one (the C component) of the three toxin components (termed A, B and C) necessary for full oral toxicity of the Tc toxins against insects [20]. The presence of Tyr-Asp repeats in TccC proteins has led others to suggest that they may bind carbohydrates [16] and could therefore be exposed at the bacterial cell surface. This hypothesis is consistent with our immuno-gold labelling experiments of Tc toxins which show that they are indeed located on the outer membrane of *Photorhabdus *bacteria [24]. Our recent work on the Tc toxins of *Yersinia *has shown that, in this more distantly related bacterium, *Yersinia *Tc's have activity against mammalian tissue culture cells [25]. It will therefore be interesting to investigate the relative toxicity of Pa and Pl Tc toxins against mammalian and insect cells to test the hypothesis that Pa Tc's are evolving towards reduced toxicity to insects and increased toxicity to mammals, like their homologues in *Yersinia*. Interestingly, we note that two exochitinase encoding genes (*pau02056 *and *pau02059*) are associated with Tc encoding loci in Pa. The presence of chitinase genes alongside those encoding these high molecular weight toxins suggests that chitinases may be used to disrupt either the peritrophic membrane (a chitinous membrane surrounding the food within the insect gut lumen) or the basal lamina (which surrounds the gut within the insect hemocoel) of the insect host in order to facilitate access of the Tc toxins to their target, the midgut epithelium.

**Table 4:** Comparative genomics of *P. luminescens *vs. *P. asymbiotica *ATCC43949 showing regions unique to each genome

**Locus**	**Species**	**Gene Region**	**Size (Kb)**	**Products of interest**
1	*P. luminescens*	*plu0179-plu0190*	8.5	Carbapenem biosynthesis operon
2	*P. luminescens*	*plu0404-plu0419*	14.3	Fimbrial proteins, adhesin
3	*P. luminescens*	*plu0525-plu0541*	16	Hemolysins
4	*P. luminescens*	*plu0545-plu0549*	16.6	Hemolysins
5	*P. luminescens*	*plu0634-plu0643*	14.2	Hemolysins, peptidase
6	*P. luminescens*	*plu0751-plu0767*	20	Proteins involved in antibiotic synthesis
7	*P. luminescens*	*plu0777-plu0793*	18	Fimbrial proteins
8	*P. luminescens*	*plu0802-plu0808*	12.8	Tc Insecticidal toxins
9	*P. luminescens*	*plu0887-plu0900*	20.7	Pyocins, proteins involved in antibiotic synthesis
10	*P. luminescens*	*plu0917-plu0925*	8	Lux-R transcriptional regulators
11	*P. luminescens*	*plu0961-plu0968*	29	Tc Insecticidal toxins
12	*P. luminescens*	*plu0991-plu0994*	3.7	Adhesin
13	*P. luminescens*	*plu1030-plu1059*	27.6	Pili operon
14	*P. luminescens*	*plu1063-plu1113*	51	Peptide synthetase, phage, transposable elements
15	*P. luminescens*	*plu1204-plu1224*	29.6	Proteins involved in antibiotic synthesis, transposable elements
16	*P. luminescens*	*plu1408-plu1413*	6.7	Hemolysins
17	*P. luminescens*	*plu1514-plu1515*	2.2	Proteins involved in tetracycline resistance
18	*P. luminescens*	*plu1536-plu1537*	1.3	Similar to Bt Insecticidal toxin
19	*P. luminescens*	*plu2188-plu2200*	14.1	Proteins involved in antibiotic synthesis
20	*P. luminescens*	*plu2213-plu2223*	15.3	Nematicidal protein
21	*P. luminescens*	*plu2903-plu2960*	33.6	Phage
22	*P. luminescens*	*plu3124-plu3129*	6.2	Proteins involved in toxin secretion
23	*P. luminescens*	*plu3423-plu3489*	47.5	Phage
24	*P. luminescens*	*plu3520-plu3535*	46.5	Proteins involved in antibiotic synthesis
25	*P. luminescens*	*plu3667-plu3669*	2.4	RTX toxins
26	*P. luminescens*	*plu3915-plu3925*	13	Proteins involved in antibiotic synthesis
27	*P. luminescens*	*plu4165-plu4175*	15.3	Tc Insecticidal toxins TccB1, TccA1, pyocins
28	*P. luminescens*	*plu4182-plu4186*	9.1	Tc Insecticidal toxins TccC6
29	*P. luminescens*	*plu4187-plu4197*	11.4	Anthraquinone biosynthesis
30	*P. luminescens*	*plu4234-plu4243*	10.5	Photopexin A & B
31	*P. luminescens*	*plu4488*	2.8	Insecticidal toxins TccC7
32	*P. luminescens*	*plu4621-plu4630*	14.6	Siderophore biosynthesis operon
33	*P. luminescens*	*plu4812-plu4830*	17.1	Lipopolysaccharide biosynthesis
34	*P. asymbiotica*	*pau00446-pau00458*	21.2	Hemolysin, Proteins involved in antibiotic synthesis
35	*P. asymbiotica*	*pau00704-pau00699*	8.3	Fucose operon
36	*P. asymbiotica*	*pau00881-pau00897*	23.6	Tc Insecticidal toxin TccC3
37	*P. asymbiotica*	*pau00911-pau00921*	18.7	Fatty acid biosynthesis operon
38	*P. asymbiotica*	*pau01194-pau01201*	16.8	Proteins involved in antibiotic synthesis
39	*P. asymbiotica*	*pau01335-pau01351*	20.1	RTX toxins
40	*P. asymbiotica*	*pau02124-pau02142*	16.3	Insecticidal toxin TccZ
41	*P. asymbiotica*	*pau02212-pau02264*	88.7	Syringomycin synthetase, insecticidal toxin, lectin
42	*P. asymbiotica*	*pau02551*	4.6	Similar to syringomycin synthetase
43	*P. asymbiotica*	*pau04327-pau04342*	18.3	Lipopolysaccharide biosynthesis

The Pa genome also shows a reduction in another class of anti-insect virulence factors, the *Photorhabdus *Virulence Cassettes or PVCs (Table 4). PVC cassettes are phage-like elements in *Photorhabdus *genomes that encode a structure similar to an R-type pyocin [26]. Each PVC cassette has several phage-like ORFs that encode the structural part of the PVC and then one or more ORFs encoding putative toxins. PVC *pnf *from Pa destroys insect blood cells [26] and we speculate that the PVCs act like a syringe to deliver the encoded effector molecules to their target cells. The Pl TT01 genome has a total of six PVC cassettes, while Pa ATCC43949 genome only encodes five. Three of these PVCs are common to both strains, Pa *PVClopT*/*PVCtt01_lopT*, Pa *PVCcif*/*PVCtt01_cif *and Pa *PVCphx/PVCtt01_4*, while Pa ATCC43949 encodes a further two unique PVCs: Pa *PVClmt *and Pa *PVCpnf*. Pl TT01 encodes four PVC elements in a tandem repeat arrangement (*plu1646-1669 *(*PVCtt01_4*), *plu1670-1689 *(*PVCu3/PVCtt01_3*), *plu1690-1709 *(*PVCtt01_2*) and *plu1710-1730 *(*PVCtt01_1*) between a type IV pilus DNA conjugation locus and a replicon partitioning gene (*mukB*). There is only one ancestral element Pa *PVCphx *(*PVCtt01_4 *homologue) at this locus in Pa ATCC43949 so it appears that it has simply failed to acquire the other three PVCs found in Pl TT01. The *PVCphx *element, which is ancestral to both Pa and Pl, is found adjacent to a type IV DNA conjugation pilus encoding operon. Similarly the equivalent virulence cassette in *Serratia entomophila*, termed the Anti-Feeding Prophage, is also found close to a type IV DNA conjugation pilus operon on the conjugative *pADAP *plasmid [27]. This close association of the conjugation pilus with PVC-like cassettes in these two widely separated groups of bacteria suggests that DNA conjugation may be responsible for the transfer of these cassettes between bacterial species.

**Figure 9:**
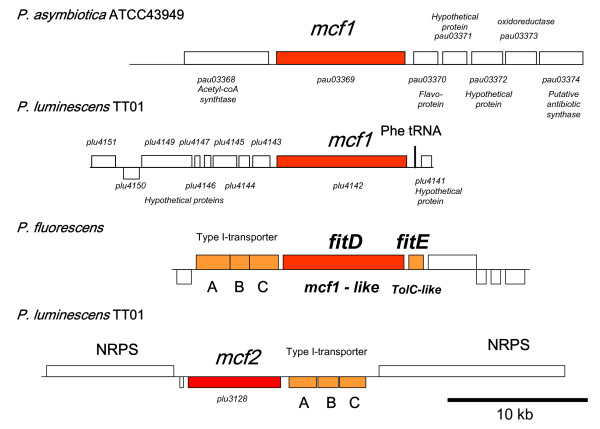
**Diagram comparing the genomic context of *mcf1 *and *mcf2*-encoding islands in Pa ATCC43949, Pl TT01 and *Pseudomonas fluorescens***. Genes similar to *mcf1 *are found in all *Photorhabdus *strains and also in the plant-associated bacterium *P. fluorescens *where the *mcf1*-like toxin encoding gene is called *fitD*. Note that *fitD *in *P. fluorescens *and *mcf2 *in Pl TT01 are encoded adjacent to ABC transporters, suggesting that type 1 secretion may be responsible for release of the Mcf-like toxin from the bacterial cell.

Consistent with further loss of insecticidal genes, Pa ATCC43949 only carries one Makes Caterpillars Floppy gene, an *mcf1*-like gene [28] whilst both Pl TT01 and Pl W14 carry the additional *mcf2 *gene [29]. The Mcf1 toxin has been shown to destroy insect phagocytes and to cause the insect midgut to disintegrate via apoptosis [28], causing the characteristic 'floppy' phenotype of *Photorhabdus *infected insects whose gut has therefore collapsed. In Pa, the *mcf1 *(*pau03369*) gene is encoded in a different locus to that of the Pl strains (Figure 9). Interestingly the *mcf1 *homologue of Pt K122 is also in a different locus again. This suggests either high motility in the genome or multiple independent acquisition of this important potent toxin in the different *Photorhabdus *species. We note that *mcf2 *in Pl is encoded next to a type I secretion system operon. Further, Mcf1 and Mcf2 both encode C-terminal domains supporting their export by a Type I secretion system. Four different Pl hemolysin-encoding loci, ranging in size from 6.7 to 16.6 kb, are also absent from the Pa genome (Table 2). The role of the extensive number of hemolysins in Pl is unclear but the loss of several hemolysin encoding loci in Pa suggests that hemolysin diversity is maintained to provide activities against a wide range of insect hosts. Finally, we note that the Pa genome also lacks a large gene lost within the deletion that removes *plu2213-plu2223 *(Table 2). This gene (*plu2222*) encodes a protein with homology to proteins listed as nematicidal proteins in GenBank but for which we can find no primary reference describing their nematicidal activity. Again, if true, this suggests that as Pa increases its virulence to man that it may be loosing anti-invertebrate virulence factors. Previous studies have suggested that variation in the toxicity of different *Photorhabdus *strains to the model nematode *C. elegans *are associated with the presence or absence of *tcdA4 *from the *tcd *pathogenicity island [30]. However, to our knowledge, no direct toxicity of Tc toxins to *C. elegans *has been demonstrated. Finally, we note that Pa ATCC43949 only has a single locus encoding an insecticidal PirAB binary toxin, unlike Pl TT01 which has two. The PirAB toxins were originally speculated to have Juvenile Hormone (JH) esterase activity and therefore to potentially interfere with development of the insect host [16]. These toxins, however, lack JH esterase activity [31] but are powerful insecticides active against both Diptera (mosquito larvae) and Lepidoptera (moth larvae) [16,31], whose mode of action remains obscure. Despite this reduction in the diversity of genes encoding insecticidal toxins, we stress that the pathogenicity of Pa to model insect hosts is in fact higher than that of Pl or Pt strains [32]. This is consistent with the hypothesis that loss of genes is not always associated with decreased virulence and in *Mycobacterium tuberculosis *exactly the opposite is true and gene deletion can often lead to hypervirulence [33].

### Novel secretion systems

**Figure 10:**
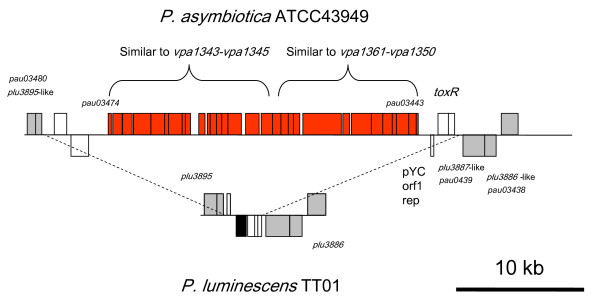
**The genome of Pa ATCC43949 carries an additional Type Three Secretion System encoding island (T3SS2) that is not present in Pl TT01**. This T3SS2 island carries two blocks of genes similar to those found in clinical isolates of *Vibrio parahaemolyticus*, the first similar to *vpa1343-1345 *and the second similar to *vpa1361-1350*.

One of the most striking differences between the insect pathogen Pl and the emerging human pathogen Pa are changes associated with Type Three Secretion Systems (TTSSs). In Pl TT01 the effector protein LopT is encoded within the single TTSS encoding operon. This LopT-like effector has been shown to inhibit the phagocytosis of Pl following its translocation by the TTSS into hemocytes [34]. In Pa this *lopT *homolog is absent from the equivalent TTSS island, however it does contain a gene previously termed *lopU *(*pau01043*) that is similar to the ExoU effector from *Pseudomonas aeruginosa *[35]. ExoU has phospholipase activity that disrupts epithelial and macrophage cell lines [36] and in Pa it may therefore have activity against human macrophages. ExoU has also been implicated in the TTSS-mediated killing of amoeba that graze *P. aeruginosa *biofilms [37,38] raising the interesting possibility that *Photorhabdus *may also use its TTSS to kill amoeba invading its infected hosts. Encoded elsewhere in the Pa genome, and potentially exported by the same TTSS, is a homolog of *sopB *(*pau01919*). SopB is important in 'directing traffic' in the early stages of *Salmonella enterica *serovar Typhimurium entry into host cells. Specifically, SopB allows the bacterium to control maturation of the *Salmonella*-containing vacuole by modulating its interaction with the endocytic system [39]. The presence of a SopB homolog in the Pa genome supports our observation that Pa bacteria can enter, and subsequently escape, macrophage cell lines (our unpublished data). Indeed clinical data, such as the bacteraemia dissemination within the body of patients, and the type of antibiotics required to control infection [40], support the hypothesis that Pa may escape the vertebrate immune system by taking refuge in macrophages in the early stages of infection. Finally, Pa has acquired a second TTSS island (Figure 10), similar to a system from *Vibrio parahaemolyticus *(see Table 5 for list of top BLAST hits in this new TTSS), here termed T3SS2, which is only found in clinical *V. parahaemolyticus *isolates [41]. The gain of this secretion apparatus in Patherefore suggests that this new TTSS may also be important in virulence against humans. In the case of *Salmonella typhimurium *infections, one TTSS operon (*spi1*) is used for delivery of effectors for initial entry into host cells whereupon the activity of the second TTSS operon, (*spi2*) delivers effectors required for intracellular persistence [42]. We speculate that the acquisition of the *sopB *effector gene and the second TTSS island have been important for the evolution of human pathogenicity in Pa.

**Table 5:** Homology of the new TTSS system with proteins in GenBank

**Locus tag**	**Protein**	**Organism**	**Score**	**E-value**
*pau03443*	Hypothetical Protein YPK_0252	*Yersinia pseudotuberculosis*	44.3	0.004
*pau03444*	hypothetical protein A55_B0283	*Vibrio cholerae*	97.1	5.00E-019
*pau03445*	putative adhesion protein	*Vibrio parahaemolyticus*	152	1.00E-035
*pau03446*	hypothetical protein A55_B0283	*Vibrio cholerae*	263	5.00E-069
*pau03447*	hypothetical protein A59_1855	*Vibrio cholerae*	77	5.00E-013
*pau03448*	peptidoglycan-associated lipoprotein	*Vibrio cholerae*	251	1.00E-065
*pau03449*	putative adhesion protein	*Vibrio parahaemolyticus*	378	9.00E-104
*pau03450*	putative type III secretion system apparatus protein VcrD2	*Vibrio parahaemolyticus*	686	0
*pau03451*	hypothetical protein A55_B0283	*Vibrio cholerae*	157	2.00E-037
*pau03452*	hypothetical protein A55_B0283	*Vibrio cholerae*	169	6.00E-041
*pau03453*	putative adhesion protein	*Vibrio parahaemolyticus*	87	5.00E-016
*pau03454*	hypothetical protein VPA1359	*Vibrio parahaemolyticus*	164	2.00E-039
*pau03455*	hypothetical protein VCB_002823	*Vibrio cholerae*	107	5.00E-022
*pau03456*	hypothetical protein A55_B0283	*Vibrio cholerae*	169	8.00E-041
*pau03457*	putative type III secretion system translocon protein VopB2	*Vibrio parahaemolyticus*	278	9.00E-074
*pau03458*	putative adhesion protein	*Vibrio parahaemolyticus*	239	8.00E-062
*pau03459*	putative adhesion protein	*Vibrio parahaemolyticus*	253	4.00E-066
*pau03460*	putative two-component response regulator	*Vibrio parahaemolyticus*	174	2.00E-042
*pau03461*	peptidoglycan-associated lipoprotein	*Vibrio cholerae*	87	5.00E-016
*pau03462*	peptidoglycan-associated lipoprotein	*Vibrio cholerae*	206	6.00E-052
*pau03463*	putative adhesion protein	*Vibrio parahaemolyticus*	88.2	2.00E-016
*pau03464*	peptidoglycan-associated lipoprotein	*Vibrio cholerae*	117	3.00E-025
*pau03465*	DNA topoisomerase I	*Vibrio cholerae*	84	4.00E-015
*pau03466*	putative type III secretion apparatus protein	*Vibrio parahaemolyticus*	101	2.00E-020
*pau03467*	ATPase, histidine kinase-, DNA gyrase B-, and HSP90-like domain containing protein	*Tetrahymena thermophila*	40.8	0.043
*pau03468*	putative adhesion protein	*Vibrio parahaemolyticus*	88.2	2.00E-016
*pau03469*	Flagellar biosynthesis/type III secretory pathway ATPase	*Vibrio cholerae*	696	0
*pau03470*	putative type III secretion system apparatus protein VscC2	*Vibrio parahaemolyticus*	639	0
*pau03471*	NO HITS	NO HITS		
*pau03472*	DNA topoisomerase I	*Vibrio cholerae*	217	3.00E-055
*pau03473*	putative type III secretion system apparatus protein VscR2	*Vibrio parahaemolyticus*	226	4.00E-058
*pau03474*	hypothetical protein A55_2001	*Vibrio cholerae*	89.7	8.00E-017

### Toxin regulation and genes expressed upon host switching

Recent work by others has employed a differential fluorescence induction approach to identify genes up-regulated in the Pl TT01 genome after exposure to insect homogenates *in vitro*. A range of toxin and toxin-related genes showed 5–10 fold fluorescence induction including the *toxin complex *gene *tccC1 *(*plu4167 *equivalent to *pau03850*). Other genes that were induced were a *photopexin *(*plu1645 *or *pau03846*) and an *RtxA*-like gene (*plu2400 *or *pau02098*). Finally *plu4122*, which contains a Fascin domain, but whose function is unknown, was also up-regulated and interestingly is also duplicated three times in the Pa genome (*pau03744, pau03746 *and *pau03747*). A second study has employed a proteomic approach to study the effects of deleting a candidate LysR-type regulator termed HcaR (*pau02358*). In this approach, *hcaR *disruption decreased expression of the insecticidal toxin genes *tcdA1*, *mcf1 *and *pirA *during exponential growth in insect hemolymph [43]. This data supports the hypothesis that LysR-type regulators are important in the regulation of insect virulence in *Photorhabdus*. Despite these elegant screens, little is known about the putative signals that either Pa or Pl uses to identify its different hosts (nematodes, insects or man). Like Pl, the Pa ATCC43949 genome contains copies of genes encoding the two global regulators, HexA (*pau01518*) and Ner (*pau03919 *and *pau04047*) which are thought to control the switch between mutualism with the nematode host and insect pathogenicity in Pl. The Pa genome also contains homologs of the two-component systems PhoQ/PhoP (*pau01733 *and *pau01734*) and AstS/AstR (*pau02265 *and *pau02266*) that have also been shown to be involved in the regulation of mutualism and pathogenicity genes in Pl, but again their role, if any, in Pa pathogenicity against humans remains unclear. Finally, the Pa genome has a multiplicity of LuxR-like receptors, which are proposed to bind host produced factors such as hormones and homoserine lactones [7]. Again possibly associated with its shift towards mammalian pathogenicity, several of these *luxR *type regulators have been lost from the Pa genome (Table 2). The Pa genomecontains 17 *luxR*-like genes, a substantial reduction from the 39 copies in Pl. The majority of the *lux-R *genes in Pl TT01 are located in two large clusters *plu0918-0925 *and *plu2001-2019*. The first one, *plu0918-0925*, is absent from the Pa genome and the *plu2001-2019 *cluster, which contains 18 *luxR*-like genes in Pl TT01, has been reduced to only six copies (*pau02572-pau02577*). Fourteen of the *lux-R *genes in Pa contain a PAS4 domain. The PAS domain appears in archaea, eubacteria and eukarya, and may play a role in insect infection by sensing insect juvenile hormone [7], a hormone that controls insect development, and could potentially enable the bacterium to adapt its gene expression to that developmental stage of the insect host. Finally, another lux-R receptor, encoded by *pau00087*, also contains a signal receiver domain; originally thought to be unique to bacteria and found in the proteins CheY, OmpR, NtrC, and PhoB, this domain has now also recently been identified in eukaroytes. This domain receives the signal from membrane located sensor partner in a two-component system (*pau00086*) and therefore also presumably plays a role in sensing the environment in the insect, nematode or indeed human, host.

### Surviving the insect immune system

Following the release of *Photorhabdus *cells from their nematode vectors, the bacteria are immediately at risk from the immune system of their new host, either insect or human. One of the main fast acting responses of all innate immune systems is the production of antimicrobial peptides or AMPs. We have recently shown that insects pre-immunized with non-pathogenic *E. coli *are subsequently able to withstand *Photorhabdus *infection and that this 'immunization' is due to elevated levels of circulating AMPs following pre-infection [44]. It has therefore been suggested that *Photorhabdus *are not inherently resistant to the humoral immune response but that the bacteria can somehow adapt to increasing levels of AMPs after infection, perhaps by altering the LPS component of the outer bacterial membrane [45]. In *Salmonella*, LPS modifications are regulated by the two component pathway PhoPQ [46] and deletion of *phoP *in Pl leads to a mutant that is both avirulent and also sensitive to the AMP polymyxin B [47]. The Pa genome still carries a *phoPQ *(*pau01734 *and *pau01733*) homolog but the role of this two component pathway in evading the vertebrate immune system remains unclear. *Photorhabdus *cells are also recognized by the insect hemocytes which attempt to phagocytose them [48]. The role of TTSS effectors delivered into insect hemocytes has been discussed above, but *Photorhabdus *also employs other pathways in an attempt to modulate nodulation, the process whereby the hemocytes encapsulate the invading bacteria in a nodule containing melanin. This process involves the production of compounds that inhibit host phospholipase A2, the enzyme involved in activation of the insect eicosanoid signalling pathway [49] which has recently been shown to be important in hemocyte migration [50]. Nodulation may also be modulated by production of the small molecule antibiotic 3,5-dihydroxy-4-isopropylstilbene (ST) which inhibits the activity of phenoloxidase, an enzyme involved in maturation of the nodule. Like Pl, Pa also makes ST (Helge Bode, personal communication), as suggested by the conservation of all the relevant biosynthetic genes in the Pa genome. Interestingly Pa also produces derivatives of ST but the role of ST or these derivatives, if any, in modulating the vertebrate immune system remains to be proven.

### Antibiotics, polyketide synthases and bacteriocins

Pl TT01 has twenty two regions encoding polyketide synthases (PKSs), non-ribosomal peptide synthases (NRPSs) or PKS-NRPS chimeras [16]. Despite this astonishingly high diversity of loci making small molecules or peptides, which make up 6% of the Pl TT01 genome, only three have been characterized in any detail. These three loci make a carbapenem antibiotic [51], an anthraquinone pigment [52] and the ST antibiotic [53]. Whilst Pa can still make the ST antibiotic, both of the loci encoding the carbapenem antibiotic and the anthraquinone pigment have been deleted from Pa (Table 4). The biological role of either of these lost loci is unclear but antibiotics have been speculated to play a role in keeping the insect cadaver clear of invading micro-organisms. The loss of these loci in Pa may therefore be another reflection of the loss of insect associated genes on its way to becoming a pathogen of man. Whilst the biological role of most of the PKS derived molecules remain obscure, it has been demonstrated that a phosphopantetheinyl (Ppant) transferase homolog, encoded by the *ngrA *gene (*plu0992*), is required for nematode association in Pl. Ppant transferases catalyze the transfer of the Ppant moiety from coenzyme A to a holo-acyl, -aryl, or -peptidyl carrier protein required for the biosynthesis of fatty acids, polyketides or nonribosomal peptides. It has therefore been speculated that the *ngrA *gene (*plu0992 *and *pau00970*) is required in the biosynthesis of a small molecule that regulates nematode development [54]. The retention of a *ngrA *homolog in Pa (*pau00970*) is therefore consistent with the recent demonstration that Pa still retains its nematode vector. Many of these PKS/NRPS loci have also been shown to make *E. coli *toxic to a range of invertebrates in recent gain of toxicity screens of a Pa ATCC43949 genomic library [55]. As well as making small molecule antibiotics, *Photorhabdus *bacteria make a range of antibacterial proteins or bacteriocins, which in *Photorhabdus *are termed lumicins [56]. S-type pyocins are composed of pairs of killer and immunity proteins and are often found in specific strains of bacteria that colonise specific niches, such as uropathogenic *E. coli*. In Pl W14, the lumicin encoding loci predict killer proteins and multiple dual type immunity proteins with domains similar to both pyocins and colicins [56]. The role of the pyocin-like loci that are incorporated into the *Photorhabdus *genome is not clear but they correspond to regions recently acquired on integrated plasmids. At least one R-type pyocin is encoded by the genome of Pl TT01 [57]. R-type pyocins are modified P2-bacteriophage tail-like structures that act to eliminate closely related strains. The R-type pyocin is encoded by ORFs *plu0008-plu0034 *and interestingly appears to have invertible DNA regions associated with alternative tail fibre genes which are likely to modulate and diversify its host-target specificity. A similar element is also encoded in the genome of Pa ATC43949 (*pau00006-pau00025*) although the arrangement of invertible DNA regions is different. Finally, a pyocin-like gene is also lost in the Pa deletion encompassing *plu4165-plu4175*, removing several Tc toxin encoding loci. The association of pyocin-like genes with *tc *encoding islands may support that hypothesis that *tc *islands represent plasmids that have integrated into the *Photorhabdus *genome [58]. The loss of this pyocin-like gene may therefore reflect the loss of its previous plasmid associated utility upon insertion of the plasmid into the bacterial chromosome.

### Adhesion, invasion and nematode re-association

During the course of their complex life cycles both Pl and Pa have to recognize and adhere to a range of very different biological substrates in both the nematode, the insect and in the case of Pa, humans. Recent studies have shown that the re-association of *Photorhabdus *with their infective juvenile (IJ) nematodes is even more complicated than originally supposed and that it involves the recognition of a number of specific tissues and cells within the nematode itself [8]. Originally, it was thought that the new generation of IJs retained *Photorhabdus *within their guts directly from within the infected insect cadaver. However more detailed examination has shown that the adult hermaphrodite nematode allows some bacteria to enter the gut and bind to a specific set of cells, the INT9 gut cells. These bacteria then infect the neighbouring rectal gland cells where they replicate inside vacuoles. In the meantime, all the IJs develop inside the hemocoel of the adult hermaphrodite in a process known as *endotokia matricida*, in which the eggs hatch internally and the emerging IJs use their mother as a food source. Finally, the infected rectal glands of the adult hermaphrodite rupture releasing *Photorhabdus *cells into the body cavity of the mother. Each developing IJ is then colonized by a single bacterium which attaches to the pre-intestinal valve cell and replicates to give a final population of around 100 cells per IJ [8]. This incredible life cycle within the nematode relies upon the successful recognition of, entry into and survival within a range of specific cell types. Unfortunately we do not know which of the numerous Pl genes encoding fimbriae, adhesins and pili are responsible for each recognition step, despite the demonstration that fimbrial-encoding loci are variable between different *Photorhabdus *isolates and may therefore be involved in the specificity of bacteria-nematode associations [59]. We do however know that Pa still retains a Heterorhabditid nematode vector, therefore we might infer that the four Pa deletions covering fimbrial proteins, adhesins and an operon encoding a pilus (Table 4) may not be involved in nematode re-association but could be losses associated with a move away from specific insect hosts. Finally, only one locus has so far been demonstrated as being required for nematode transmission, that is the Pl *pgbPE *operon (*plu2654-plu2660*) [60] corresponding to *pau01881-pau01875*. The *pgbPE *operon is required both for insect pathogenicity and nematode mutualism, and is also involved with resistance to AMPs. As the ability to resist AMPs is important in the persistent infection of *Caenorhabditis elegans *by *S*. *e*. Typhimurium, this had led Clarke to speculate that *Photorhabdus *may also have to overcome the humoral immune response of its nematode host [45].

### Emerging into the human immune system

Australian isolates of Pa have recently been shown to be vectored by nematodes and although unproven for Pa in North America, nematode vectoring remains the most likely route of infection for the patient(s) discussed here. Unlike Pl, therefore Pa bacteria also have to survive the human immune system following their presumptive release by IJs infecting either human wounds or direct entry through un-perforated skin. As discussed earlier the link between human pathogenicity and the presence of *pPAU1 *like plasmids suggests that they may encode genes relevant to human infection. Attempts to cure Pa ATCC43949 of its plasmid through growth at elevated temperature have not been successful, suggesting it plays an important role in Pa, even *in vitro*. It is obvious that the ability of Pa to survive and grow at 37°C is essential for the human pathogenicity of Pa and a proteomic comparison of cells and supernatants from Pa grown at 30°C and 37°C revealed that at 37°C two heat shock proteins are induced. These are the ClpB-homologue *pau03190 *and the HtpG-homologue *pau03384 *(homologues in Pl TT01 to *plu1270 *and *plu3837 *respectively). In addition to the temperature increase upon entry into a human, Pa must also resist the fast acting innate immune response. In this respect, it is interesting to note that a small protein with homology to the attachment invasion locus protein Ail from *Yersinia pestis *is also secreted at 37°C by Pa but not at 30°C and that in *Y. pestis *Ail gives resistance to human complement [61] (Figure 2).

**Figure 11:**
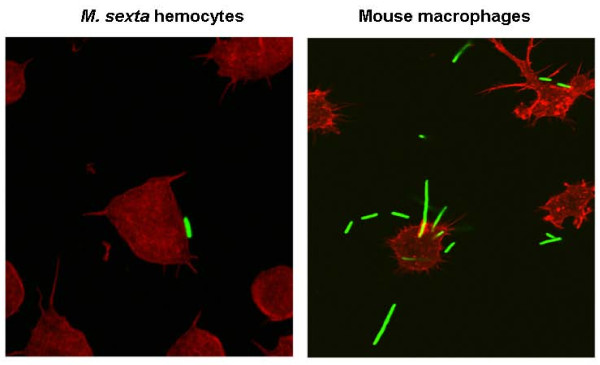
**Difference in the behaviour of GFP labelled Pa ATCC43949 bacteria when exposed to insect hemocytes from *Manduca sexta *(left panel) and mouse-derived macrophage cell lines (right panel)**. Note that Pa cells merely adhere to the surface of the insect phagocytes whereas in the presence of macrophages the bacteria grow as filaments that protrude from and invade the mouse phagocytes. Phagocytes are labelled with a TRITC-phalloidin conjugate to visualize their actin cytoskeletons.

**Figure 12:**
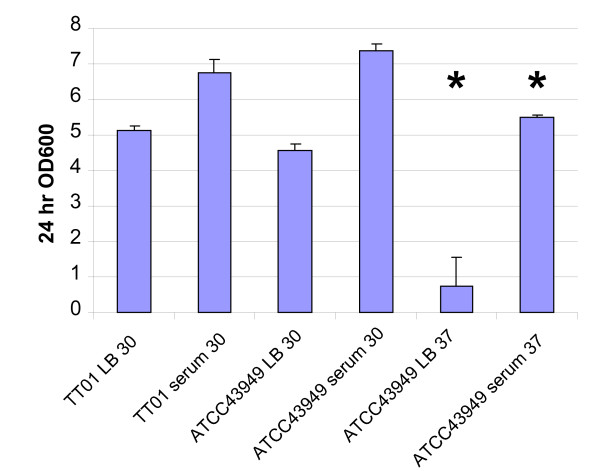
**Growth of Pl TT01 and Pa ATCC43949 LB medium in the presence or absence of human blood serum at 30°C and/or 37°C**. Note that Pa ATCC43949 experiences a long lag phase when grown at 37°C in LB which is abolished by the addition of human serum. The asterisks denote that bacterial cells grew as filaments at 37°C in a fashion similar to their growth in the presence of mouse macrophages (see Figure 11).

In addition to changes in protein profiles, it is possible that Pa may also modify the structure of the outer membrane lipopolysaccharide (LPS) upon human infection, a common strategy used by Gram-negative pathogens to avoid recognition. A comparison of the LPS-biosynthesis genes of Pl TT01 and Pa ATCC43949 shows an extensive region which is different between the two strains. A large genomic region between *plu4796-plu4811 *and *plu4831-plu4862 *is common, with the exception of one or two genes. However the Pl TT01 region from *plu4813-plu4830 *is absent from Pa ATCC43949 and in the place of this 17 kb region is an 18 kb region encoding gene homologues involved in O-antigen synthesis from a range of other bacteria (*pau04327-pau04342*). We speculate that acquisition of these genes is important in mammalian virulence. In addition to changes in LPS, the deployment of extracellular polysaccharide (EPS) is also often important in infection. Interestingly *in vitro*, Pa does not readily form biofilms in static liquid culture at 37°C, but will do so at 28°C. This suggests a significant difference of CPS/EPS deployment at human and insect relevant temperatures resulting in a differential ability to perform biofilms in the two different conditions. Finally, confocal microscopy of *in vitro *tissue culture experiments in which we challenged mouse macrophages with Pa ATCC43949 revealed that bacteria were either internalised or invaded macrophages rapidly but were then capable of re-emerging from the cells by growing in a filamentous form (Figure 11). This correlated with gentamycin exposure assays that confirmed that the Pa cells were protected from the extracellular antibiotic for 8 h post exposure but then again became exposed and susceptible. This ability to colonize macrophages may be important in establishing early Pa infections in mammals shortly after their release from their vector nematodes. In contrast, when presented with insect hemocytes, Pa cells adhered to the outside of the insect phagocytes and did not invade or replicated within them (Figure 11). Finally, to test the hypothesis that Pa is specifically adapted to growth in humans, we compared the ability of Pl and Pa strains to grow at 30°C and 37°C in LB medium in the presence and absence of human blood serum. When grown at 37°C in LB, Pa ATCC43949 shows a long lag phase in its growth which can be removed by the addition of human blood serum (Figure 12). This suggests that Pa has evolved a specific ability to grow in human blood at elevated (mammalian) temperatures.

## Discussion

### The relationship of Pa ATCC43949 to other *Photorhabdus*

The genus *Photorhabdus *is currently divided into three species [62]. *P. luminescens *(Pl) and *P. temperata *(Pt) are exclusively associated with nematodes that kill insects, whereas, until recently, strains of the third species *P. asymbiotica *(Pa) have only been recovered from human wounds [5,6]. This situation has now changed with our recent description of a nematode vector for an Australian isolate of Pa, suggesting that all Pa strains do indeed have nematode symbionts, contrary to the a-symbiotic lifestyle suggested by their specific name [15]. This suggestion is also consistent with recent independent reports of Pa strains associated with vector nematodes in Japan [63]. In order to look at the changes in genome structure and composition associated with the ability to infect man, we have chosen to sequence one of the North American isolates of Pa, specifically ATCC43949. Confusingly, in the original 1989 paper describing the isolation of this strain from an 80-year-old female patient with endocarditis in Maryland, this strain is described as *Xenorhabdus luminescens *strain 2 which was ascribed to *X. luminescens *DNA hybridization group 5 [5]. Subsequently the classification of the genus *X. luminescens *was changed to *Photorhabdus luminescens *(*Photo*- meaning light and -*rhabdus *meaning rod). The correct classification of the strain whose genome has been described here is therefore *Photorhabdus asymbiotica *strain ATCC43949 or simply, Pa ATCC43949.

### How many immune systems does Pa need to evade?

When the Pl and Pt lifecycles were first described it was thought that only the immune system of insects would challenge the growth of *Photorhabdus *in their different hosts. Now, with the new description of *endotokia matricida *as the likely standard method of nematode reproduction [8] it seems that successful re-association with the IJ nematodes also involves exposure to the immune system of the nematode host. With the emergence of Pa, this third *Photorhabdus *species now has to cope not only with two different invertebrate immune systems but also with a third immune system, that of man. In this light we can now re-examine the lifecycle of Pl and Pa and ask what virulence factors are likely to be used by which bacterium at which stage and in which host. Specifically, if Pl has to face the insect immune system after being regurgitated by the IJ nematode, what factors does it use to overcome the subsequent up-regulation of AMPs and the presence of circulating phagocytic hemocytes? Subsequently, if Pl also has to face the immune system of its nematode host upon the release of bacteria from cells within the nematode gut into the nematode hemocoel, how does it evade or overcome the nematode humoral immune response? Finally, if Pa is indeed released into humans after regurgitation by invading IJs, how does Pa evade or overcome the different aspects of the human immune response, which now also involve antibody based responses? By comparing the genomes of Pl and Pa we can therefore ask which "tools" *Photorhabdus *is likely to use against specific invertebrate or vertebrate hosts, and also which tools are likely to be useful against both classes of host? In other words, are the insect and nematode immune system functionally equivalent and what changes does *Photorhabdus *have to make to move from resisting an invertebrate immune system to dealing with a human immune system. Previous attempts to compare the genomes of Pa strains with entomopathogenic *Photorhabdus *species have been restricted to genomic subtractive hybridization techniques [64]. Genomic subtraction between two different Australian Pa strains and several different Pl strains has previously revealed specific differences in encoded virulence factors, such as SopB [64], but here we have compared the full genomic sequence of one specific USA Pa isolate ATCC43949 with the entomopathogenic Pl strain TT01, and can therefore derive an exhaustive list of all the genomic differences observed.

### Changes in the diversity of insecticidal toxins

When the first *Photorhabdus *genome was sequenced in 2003 by the Institute Pasteur (France), the genome of Pl TT01 was described as carrying more toxins and proteases, candidate anti-insect virulence factors, than any other bacterial genome sequenced to date [16]. It has therefore been very informative to examine which of the different classes of known insecticidal toxins have been reduced or lost in the evolution of Pa ATCC43949. Most notable are changes in the number of copies of the insecticidal *tc *genes. The Toxin complexes (Tc's) are orally active insecticidal toxins, transformed both into transgenic plants [65] and most recently into recombinant termite gut bacteria [66] for insect control, which are encoded at several different genomic islands in *Photorhabdus *genomes. Pa ATCC43949 shows loss of *tc *genes in both the Tca and Tcd encoding gene islands suggesting a potential reduction in its oral toxicity to insects. These observations are consistent with our earlier genetic work in Pl strain W14 which showed that deletion of either the *tca *or *tcd *island reduced oral toxicity of the bacterial supernatant to *M. sexta *larvae and that deletion of both loci abolished oral activity altogether [21]. We therefore speculate that the loss of copies of *tca *or *tcd*-like genes at these two genomic locations in Pa ATCC43949 is the reason for the absence of oral toxicity to model insects. This hypothesis, however, remains to be tested. For other classes of anti-insect virulence factors such as the *Photorhabdus virulence cassettes *or PVCs [26], changes in both the numbers and composition of PVC cassettes present can be seen. Specifically, Pa ATCC43949 again shows a reduction in the total number of copies of these insecticidal genes and the nature of the candidate virulence factors encoded within the 'pay-load' region of each PVC cassette has also changed. In previous studies we have shown that the effector encoded by Pa PVC *pnf *destroys insect phagocytes [26] and we speculate that the PVCs act like a syringe to deliver the encoded effector molecules to their target cells. It would therefore be interesting to examine if the PVC encoded effectors of Pa have any effects on human macrophages. Despite the reduction in the number and type of insecticidal toxins encoded in the Pa genome, the pathogenicity of Pa to model insects remains higher than that of either Pl or Pt [32]. This observation is hard to explain unless either, Pa has a more restricted insect host range than Pl or Pt and therefore needs a reduced number of toxins to kill a narrower range of insects, or, and perhaps more interestingly, Pa is similar to bacteria like *M. tuberculosis *in which gene deletion actually leads to increased, rather than decreased, virulence [33].

### Gain of pathogenicity to man

In order to develop an assumption free approach to the functional annotation of the Pa genome we have recently developed a new screening approach, termed Rapid Virulence Annotation or RVA [55]. This technique based on the parallel screening of genomic DNA libraries against a range of both invertebrate (insects, nematodes and amoeba) and vertebrate (macrophage) targets. In a recent study we have used this technique to screen a cosmid library from Pa ATCC43949 against this range of diverse targets and to identify clones encoding virulence factors via their gain of toxicity to, or persistence within, the target cells or hosts [55]. Interestingly, and perhaps surprisingly in the current context, most of the virulence factors identified were active against most of the taxa [55]. For example there were very few factors that were active against macrophages but not against insects. This broad, assumption free, screen of Pa virulence factors supports the concept that *Photorhabdus *virulence factors are as effective against invertebrates as they are against vertebrate cell lines such as macrophages. Therefore, this suggests that a few functional changes, such as the gain of a plasmid, may be responsible for the ability of Pa to infect man. Extensive screens of both Pl and Pa strains were made here in order to look for the presence of plasmids. Despite earlier reports of plasmids in Pl strains by other authors [67], in this study plasmids were only ever recovered from Pa strains suggesting that their acquisition is indeed key to their ability to infect man (Figure 1).

The insect pathogen Pl resists its own phagocytosis by hemocytes using TTSS delivered effectors. In contrast, Pa appears to invade macrophages, probably enabled by the acquisition of the second TTSS (here termed T3SS2, Figure 10) and the novel SopB-like TTSS delivered effector, both of which may facilitate the persistence of Pa cells within macrophages. This observation agrees with other recent data describing the ability of both Australian and North American Pa strains to replicate in macrophage-like tissue culture cells where they have also been shown to cause apoptosis [68]. Apoptosis in macrophages is probably associated with the dominant apoptotic toxin Mcf1, a gene for which is present in the genome of Pa, which causes mitochondrially mediated apoptosis in a wide range of cell lines including macrophages [69]. As well as actually invading macrophages, growth at the human body temperature of 37°C causes Pa cells to adopt a filamentous mechanism of growth and division (Figure 11). The precise mechanism whereby this switch in Pa growth form comes about is not clear but in uropathogenic *E. coli *filamentation is controlled by the cell division inhibitor SulA and has been proposed as a mechanism of evasion of the innate immune response in murine cystitis [70]. We note that Pa ATCC43949 also shows a very long lag phase when switched from growth at 30°C to 37°C in LB medium (Figure 12). Interestingly the presence of human serum allows Pa ATCC43949 to overcome this lag in growth, suggesting it provides a signal or nutrient required for the switch to mammalian pathogenesis. We also note that neither Pl TT01 nor Pa ATCC43949 is susceptible to the rapid killing normally associated with incubation with human serum, as occurs with *E. coli *K12 controls. This suggests that at least these two strains of *Photorhabdus *are inherently resistant to complement recognition, activation and/or killing. In the case of *Burkholderia pseudomallei *the presence of the capsule is required to prevent complement factor C3b binding, an essential step in complement killing [71]. We speculate that as both insect and human hosts deploy fast acting innate immunity recognition and killing mechanisms, either complement or phenol-oxidase cascade, a common defensive barrier may be deployed by Pa which can protect against either pathway. We suggest that the Pa capsular polysaccharides may fulfil this role. In this regard, it is pertinent that both Pl TT01 and Pa ATCC43949 grown at 28°C are resistant to human serum. In other species of bacteria resistance to human complement is associated with the Attachment and Invasion (Ail) protein, a homologue of which is encoded by Pa ATCC43949. It would therefore be interesting to test the relative roles of capsular polysaccharide and Ail in the ability of Pa to grow in human sera by analysis of deletions in the corresponding loci.

### Antibiotic and pigment production

Pl TT01 carries a cluster of genes involved in the production of a carbapenem-like antibiotic [51]. Carbapenems are a class of β-lactam antibiotics with broad spectrum activity that are synthesized via a different route than the sulphur ring β-lactams such as penicillin [51]. In Pl, the production of carbapenem has been speculated to control the likely growth of the insect gut flora following their potential migration into the hemocoel of the infected insect [51]. The loss of the carbapenem encoding gene cluster from Pa ATCC43949 may again potentially reflect a reduced association with insects. Similarly, the cluster of genes encoding the anthraquinone [52] is also absent from the Pa genome. The absence of this pigment-encoding locus may therefore be responsible for the reduced pigmentation typically shown in Pa colonies versus those from either Pl or Pt. However, knock-out of the anthraquinone encoding locus in Pl TT01 resulted in no observable differences in pathogenicity to insects or the ability of the mutant to re-associate with its nematode host [52]. Therefore, beyond pigmentation, the role of the anthraquinone, and therefore the potential reasons for its loss from Pa, are still unclear. Finally, we note that although it is relative simple to predict the likely structure of these natural products of polyketide origin from the nature and order of the PKS encoding genes in the synthetic clusters, the precise chemical structure and activity of very few of these compounds has been examined [52,53]. Further elucidation of the role of PKS related gene products from different *Photorhabdus *will require a systematic isolation of these compounds and testing of their biological activities against a range of targets. In this respect we note that several hits from the RVA screen of Pa ATCC43949 were products from PKS-like loci [55] and that, despite the absence of the appropriate biosynthetic machinery in the host *E. coli*, RVA is a good technique for picking up low levels of biological activity associated with small molecules and peptides made by PKS-like loci.

### Future perspectives on *Photorhabdus *genomics

The sequencing of one of the clinical isolates of Pa from the North American subgroup now places new emphasis on a comparison with Pa strains isolated from Australia in order to understand how these two groups of infections have occurred on two widely separated continents. To this end we are using next generation sequencing technologies, both Solexa and Roche 454 based, to derive the genome sequence of Pa strain Kingscliffe, a recent Australian isolate derived from the hand of a man digging a fence post in sandy soil in the village of Kingscliffe on the Australian Gold Coast [15]. As well as examining the genomic differences associated with Pa strains from opposite sides of the globe, we are also keen to sequence the complete genome of a representative of the third species group, *P. temperata *strain K122, again using next generation sequencing technology. Such comparisons between the two different clades of Pa strains, and the other species of strict insect pathogen Pt, should enable us to understand how many different times *Photorhabdus *has evolved the ability to infect humans.

## Conclusion

The sequencing of the Pa genome of a North American isolate shows the addition of a plasmid *pAU1 *and several new pathogenicity islands including one encoding an additional type III secretion system putatively facilitating intracellular survival. The genome of Pa, which is a pathogen of both insects and man, illustrates the novel lifestyle of Pa over Pl, which is exclusively a pathogen of insects, and suggests how Pa can overcome the human immune system and persist inside macrophages. Comparison with the recently described lifecycle of Pa strains from Australia suggests that Pa strains from North America are also vectored by nematodes but at this stage this remains an assumption and therefore the search for a nematode vector for North American Pa strains is a research priority.

## Methods

### Plasmid screening and genome sequencing

Twenty strains of Pl and seven strains of Pa (four from North America and three from Australia) were screened for the presence of plasmids, as previously reported to be present in some strains of Pl [67]. For genome sequencing, a single colony of *P. asymbiotica *strain ATCC43949 was picked from LB agar and grown overnight in LB broth with shaking at 37°C. Cells were collected and total DNA (10 mg) was isolated using proteinase K treatment followed by phenol extraction. The DNA was fragmented by sonication, and several libraries were generated in *pUC18 *using size fractions ranging from 1.0–2.5 kb. The whole genome was sequenced by a shotgun approach to a depth of 11× coverage from pMAQ1b_*Sma*I (insert size 5.5–6 kb) and *pUC19 *(insert size 2.8–5.5 kb) small-insert libraries using dye-terminator chemistry on ABI3700 and ABI3730 automated sequencers. End-sequences from larger insert plasmid (pBACe3.6_*Bam*HI, 10–25 kb insert size) libraries were used as a scaffold. Sequences were assembled using the Phusion assembler [18], based on read pair information. A Phrap incremental assembly was then performed on the contigs generated by the Phusion assembly to include all reads not included in the initial assembly. Gap closure and finishing used the GAP4 genome assembly program  in combination with directed sequencing, primer walks and subcloning and shotgun sequencing of bacterial artificial chromosome clones or Polymerase Chain Reaction (PCR) products that spanned sequence gaps. An ACT alignment of Pa against Pl TT01 was used to scaffold un-bridged contigs which helped guide subsequent PCR reactions. The remaining gaps were closed by combinatorial PCRs. The finished sequence was compared with DNA sequences in public databases by homology searches using Fasta, BlastX, and BlastN [72]. Potential coding sequences (CDSs) were predicted using a combination of the *ab initio *gene prediction programs Orpheus [73] and Glimmer [74]. Manual curation was performed to verify the accuracy of gene predictions which were then searched for Pfam [75] domains, and compared with the protein databases by using BlastP. Artemis [76] was used to collate data and facilitate annotation. Stable non-coding RNAs were identified by comparison with the Rfam database [77].

### Labeling of Pa with Green Fluorescent Protein (GFP)

Pa cells were labelled with GFP via insertion of the plasmid *pBamH7*. The plasmid was delivered via conjugation with S17-1λpir by using a membrane filter mating technique. S17-1λpir *pBamH7 *was inoculated into 5 ml of LB broth containing kanamycin and grown at 37°C for 16–18 h with shaking (200 rpm). Pa was grown at 28°C for 16–18 h with shaking (200 rpm) but without antibiotic selection. 100 μl of each saturated bacterial culture was added to 3 ml of sterile 10 mM MgSO4, mixed, and filtered through a 0.45-μm-pore-size nitrocellulose filter, using a 25-mm Swinnex filter apparatus (Millipore). Control assays, using donor and recipient alone, were also performed. Filters were placed on LB plates supplemented with 10 mM MgSO4 and incubated for at least 8 h in a 37°C incubator. The filters were washed with 4 ml of sterile 0.85% NaCl, and 100 μl aliquots were spread onto LB plates containing 25 μg of rifampicin and 25 μg of kanamycin per ml. Rifampicin-resistant and kanamycin-resistant transconjugants were identified after 48 h incubation at 37°C.

### Response of Pa to temperature, phagocytes and human serum

To look at the behaviour of Pa cells to changes in temperature we performed two-dimensional (2D) analysis of cytoplasmic proteins expressed in cultures grown at 30°C versus 37°C. Supernatant preparation and 2D-gel analysis were performed as previously described for *Photorhabdus *cells [78]. To look at the behaviour of Pa cells and insect phagocytes, GFP labelled cells of Pa ATCC43949 (10^-^6 cfu/ml) were injected into 5^th ^instar *Manduca sexta *larvae and left at 25°C for 6h. 250 μl of hemolymph was then bled from each insect into 1ml of ice cold Grace's Insect Medium (GIM). The resulting cell suspension was centrifuged at 1000 rpm for 10 min at 4°C to separate hemocytes from the plasma and then re-suspended in 250 μl of fresh ice cold GIM. 50 μl of the cell suspension was applied to glass slides and cells were allowed to adhere for 30 min. Cell monolayers were then fixed with 4% Pfa for 30 min at room temperature. For staining with TRITC-phalloidin, monolayers were washed with PBS to remove Pfa and incubated with 50mM NH4Cl for 10 min to reduce autofluorescence. Monolayers were again briefly washed with PBS and permeabilised with 0.1% Triton for 10min. Finally, cell monolayers were washed with PBS and blocked with 0.1% PBS-BSA for 30 min and incubated with 1ug/ml of TRITC-phalloidin for 20 min in the dark before microscopy. To examine the interaction of Pa cells with mouse BALB/C mouse macrophage cell line J774.2. Bacteria were applied to cell monolayers at a multiplicity of infection of 100:1 and centrifuged for 5 min at 800 rpm at room temperature. They were allowed to adhere for 2h and the supernatant was replaced with fresh pre-warmed RPMI medium without serum. Medium was replaced every hour until 6 h post infection. To determine the number of internalized bacteria a gentamicin killing assay was used to eliminate all bacterial cells not internalized, as described in detail elsewhere [68]. Finally, for the testing of resistance to human serum, Pa ATCC43949 cultures were started at 0.01 OD600 from a fresh overnight 4ml LB starter culture and grown, with shaking at 260 rpm, in the presence and absence of 1/5 v/v dilution of commercially available human blood serum (Lonza, catalogue number 14-402E).

## Authors' contributions

RffC and NW conceived of the study and drafted the manuscript. PW performed the manual annotation of the Pa genome and helped draft the manuscript. MSC and IV carried out the biological experiments to look at the growth of Pa in the presence of macrophages and human blood serum. At the Sanger Centre, Carol C, LC, NT and JP were responsible for co-ordinating the sequencing program. Craig C, AB, AB, LC, JD, DO, MM, NB, FS, MS, CA and MAR were responsible for the laboratory based preparation of clones for sequencing, for finishing the sequence and gap closure. MQ was responsible for library production and long range PCR to resolve final ambiguities in the genome assembly. All authors have read and approved the manuscript.
